# 

**Published:** 1892-07

**Authors:** 


### BOOKS RECEIVED.

Transactions of the Southern Surgical and Gynecological Association. Vol. IV. Fourth session. Held at Richmond, Virginia, November 10, 11, 12, 1891. Published by the Association. Philadelphia: Wm. J. Dornan, Printer.

Proceedings of the Philadelphia County Medical Society. Vol. XII. Session of 1891. T. B. Schneideman, M. D., editor. Philadelphia: Printed for the Society. 1891.

Manual of Skin Diseases, with special reference to diagnosis and treatment, for the use of students and general practitioners. By W. A. Hardaway, M. D., Professor of Skin Diseases in the Missouri Medical College and in the St. Louis Post-Graduate School of Medicine; Dermatologist to the Augusta Free Hospital for Children; Consulting Dermatologist to the City and Female Hospitals; Ex-President of the American Dermatological Association. Philadelphia: Lea Brothers & Co. 1891.

U. S. Department of Agriculture, Division of Chemistry. Bulletin No. 13. Foods and the Food Adulterations. Investigations made under direction of H. W. Wiley, Chief Chemist, with collaboration of

H. A. Huston, H. H. Nicholson, W. B. Rising, M. A. Scovell, S. P. Sharples, W. C. Stubbs, Shippen Wallace, F. G. Weichmann, H. A. Weber, and K. P. McElroy. Part sixth. Sugar, Molasses and Syrup, Confections, Honey and Beeswax. Published by authority of the Secretary of Agriculture. Washington: Government Printing Office. 1892.

U. S. Department of Agriculture. Division of Chemistry. Bulletin No. 32. Special Report of the Extent and Character of Food Adulterations, including State and other Laws Relating to Foods and Beverages, by Alex. J. Wedderburn, Special Agent. Published by authority of the Secretary of Agriculture. Washington: Government Printing Office. 1892.

Bureau of Education. Circular of Information No. 9, 1891. Biological Teaching in the Colleges of the United States, by John P. Campbell, A. B., Ph. D., (Johns Hopkins,) Professor of Biology in the University of Georgia. Washington: Government Printing Office. 1891.

Bureau of Education. Circular of Information No. 5, 1891. Contributions to American Educational History, edited by Herbert B. Adams. No. 12. The History of Higher Education in Ohio. By George W. Knight, Ph. D., Professor of History, Ohio State University, and John R. Commons, A. M., Associate Professor of Political Economy, Oberlin College. Washington: Government Printing Office. 1891.

A System of Practical Therapeutics. Vol. III. Edited by Hobart Amory Hare, M. D., Professor of Therapeutics and Materia Medica in the Jefferson Medical College of Philadelphia; assisted by Walter Chrystie, M. D. In three volumes, comprising 3,544 pages, with 434 illustrations. Per volume, cloth, $5.00; leather, $6.00; half Russia, $7.00. Sold by subscription only. Philadelphia: Lea Brothers & Co. 1892.

Notice to Contributors.—We are glad to receive contributions from every one who knows anything of interest to the profession. Articles designed for publication in the Journal should be handed in before the first day of the month. The Editors are not responsible for the views or opinions of contributors. All communications should be addressed to the Managing Editor, 284 Franklin St., Buffalo, N. Y.

### INDEX.

PAGE.

ABDOMINAL SECTION, Six Cases of . . . . . . . . . 710
Surgery, Recent Work in 90
Tumors Complicated by Pregnancy. How shall we proceed when they are? . . 345
Abdomen, Treatment of Penetrating Wounds of . . . . . . . . . . 160
Abscess of the Ano-Rectal Region, Diagnosis and Treatment of . . . 641
Academy of Medicine, Buffalo . . . . . . . . . . . . 549, 689, 728, 745
All Around the Year, 1892 . . . . . 553
American Lancet and the Detroit Meeting . . . . . . . . . . 681
Medical Association, Membership in . . . . . . . . . . 109
Pharmaceutical Association, Membership in . . . . . . 369
Physicians, Biography of . . . 553
Anemia, Case of Pernicious . . . . 436
Anesthetic, A New Local . . . . . 406
Anesthetics, Idiosyncrasy in Admintration of . . . . . . . . . . . . . 740
Antiseptics, Internal . . . . . . . 539
Appendicitis, A Discussion of . . . 258
Report of Cases of . . . . . 258
Four Operations for . . . . . 269
Operative Treatment of . . . 275
Aristol . . . . . . . . . . . . . . 50
For Bedsores . . . . . . . 653
Arthrectomy of the Knee-Joint . . . 1
Artists—Are they Pugilists? . . . . 101
Asepsis in Intra-Peritoneal Surgery . 420
Asthma Powder . . . . . . . . . 21

BACTERIA of the Mouth . . . 715
Barker, T. Ridgway, M. D. Does Organic Disease of the Heart Preclude the Use of Chloroform in Parturition? 649
Barnum, Eugene E., M. D. Transverse Presentations. History of a Case, with a New Method of Treatment . . . . . . . . . . . . . . 385
Benedict, A. L., M. D. Metric Formulæ . . . . . . . . . . 233
Sub-Acute Parenchymatous Hepatitis and Gastro-Enteritis . . . . . . . . . . . 522
Books and Pamphlets Received . . . 62, 125, 190, 251, 317, 380, 509, 638, . . . . . . . . . . . . . . 701, 761

PAGE.

Brain Surgery, Clinical Contributions to . . . . . . . . . . . . . . . 389
Brown, Geo. L., M. D. Duties of Physicians as Examiners for Life Insurance . . . . . . . . . . . . 69
Bubo, Treatment of . . . . . . . . . 539
Buffalo, Health Department of . . . 487
Academy of Medicine . . . . . . . . . . 549, 689, 728, 745
University, College Commencement . . . . . . . 680
Burns, Treatment of . . . . . . . . 161

CARBOLIC ACID, Destructive Action of . . . . . . . . . . 159
Cataract, Incipient . . . . . . . . . 356
Catgut Ligatures, Absorption of . . 678
Cesarean Section, Improvements in Method of . . . . . . . . . . . 230
Chemotaxis . . . . . . . . . . . 677
Child—Is it Viable at Six and a Half Months? . . . . . . . . . . . . 342
Child Marriage in India . . . . . . 491
Chloralamid in Epilepsy . . . . . . 365
Chlorine Water in Operations on the Eye . . . . . . . . . . . . . . . 589
Chloroform, Internal Uses of . . . . 540
In Parturition . . . . . . . . 649
Cholecystotomy, Report of Cases of . . . . . . . . . . . . . . 414, 415
Chorea Cured by Exalgine . . . . . 489
Church Bells, and Music in Court . . 618
Nuisance of . . . . . . . . 688
Clark, Edward, M. D. Diagnosis and Treatment of Abscess of the Ano-Rectal Region . . . . . . . . . 641
Club-Foot Shoe, A New . . . . . . 361
Cocaine and Ether Spray . . . . . 159
Fatality of . . . . . . . . . 324
Conjunctivitis, Chronic . . . . . . . 357
An Overlooked Factor in the Production of . . . . . . . 705
Consumption, Concentrated Food in the Treatment of Pulmonary . . . 74
Coroner's Juries and the Park Place Disaster . . . . . . . . . . . . 358
Correction . . . . . . . . . . . . 110

**Correspondence:**

Berlin Letter, from Dr. Frank J. Thornbury . . . . . . . . . 543

PAGE.
Crime and Responsibility . . . . . 51
Increase of, and the Death Penalty . . . . . . . . . 99
Criminal, an Infant . . . . . . . . 102
Criminals, the Proper Treatment of . 617
Crockett, M. A., M.D. Six Cases of Abdominal Section . . . . . . . 710
Corson, Hiram, M. D. Pneumonia and its Treatment . . . . . . . 656
Cushing, Clinton, M. D. Shall we Use the Uterine Sound to Correct Backward Displacements of the Uterus? . . . . . . . . . . . . 201
Cystitis, Injections for Chronic . . . 542

DAVIS, W. E. B., M. D. Recent Work in Abdominal Surgery . 90
De Schweinitz, G. E., M. D. Cases of Obstructive Disease of the Lachrymal Passages, and Associated Intra-Nasal Lesions . . . . . . . 593
Diarrhea, Prescription for . . . . . 7
Diphtheria, Paraffin in . . . . . . . 299
Ice in . . . . . . . . . . . 490
Dowd, J. Henry, M. D. A Case of Syphilodermata . . . . . . . . 330

EAR SCISSORS, New . . . . . 163
Ectopic Gestation, Moot Points in . . . . . . . . . . . 477

**Editorials:**

American Association of Obstetricians and Gynecologists 181
Medical Association at Detroit . . . . . . . . 741, 746
Appendicitis—Discussion in the Philadelphia County Medical Society . . . . . . . . . . 303
Buffalo Academy of Medicine . . . . . . . . . . . 549, 689, 745
Civil Service Examination for Physicians . . . . . . . . . 56
College Commencements . . . 679
Current Topics . . . . . . . . 624
Drawer, Editor's . . . . . . . 551
Editorial Retirement of Dr. Mundé . . . . . . . . . . . 363
Empire State Express . . . . . 302
Exempts, The . . . . . . . . 183
Ex-Health Officer William M. Smith . . . . . . . . . . . 744
Frank Hamilton Potter, M. D. . 53
Important to Medical Students . 109
Koch vs. Virchow . . . . . . . 108
Medical Mecca, A . . . . . . 184

PAGE.
Medical Examining and Licensing Board, State . . . 179
Practice in Connecticut . . . 182
Society of the County of Erie . . . . . . . . . . . 744
Society of the State of New York . . . . . . . . 127, 496
Societies of America, General and Special . . . . . . 193
Melange, Editorial . . . . . . 440
Mississippi Valley Medical Association . . . . . . . . . . . 236
National Medical Benevolent Fund . . . . . . . . . . . 623
New York Physicians' Mutual Aid Association . . . . . 550, 745
Ohio Moves for State License . 365
Opium Treatment of Peritonitis . 182
Pan-American Medical Congress . . . . . . . . . . . 235, 551
Physician in Politics, The . . . 439
Sanitary Condition of Public Schools . . . . . . . . . . . 364
Sheppard Hospital for the Insane 177
Southern Surgical and Gynecological Association . . . . . 300
State Medical Examining and Licensing Boards, National Conference of . . . . . . . . 747
Street Cars and the Public Health 362
Students of Niagara University Speak . . . . . . . . . . . 498
The Late Dr. F. H. Potter . . . 164
Topics of the Month . . . . 681–743

Electricity—Is it Manufactured? . . 489
Epilepsy, Chloralamid in . . . . . . 365
Ether Spray and Cocaine . . . . . . 159
Extremities, Asymmetry of . . . . . 606
Eye, Chlorine Water as an Antiseptic in Operations and Injuries of the . . . . . . . . . . . . . . 589

FARADIC COIL in Gynecological Practice . . . . . . . . . 465
Femoral Adduction, Abduction, and Flexion . . . . . . . . . . . . . 531
Fever, Enteric, Treatment of . . . . 539
Food Exposition, 1892 . . . . . . . 440
Bad and Malnutrition . . . . 456
Manufacturers' Association . . . . . . . . . . . . . 625, 743
Frederick, C. C., M. D. Operative Treatment of Retro-displacements of the Uterus . . . . . . . . . . 577
Fresh Air Mission, and Cholera Infantum Hospital . . . . . . . . . 689
Fund, A National Medical Benevolent . . . . . . . . . . . . . . 623

PAGE.
GALL STONES, Sweet Oil in the Treatment of . . . . . . . . 207
Gangrene, Senile . . . . . . . . . . 457
Gas, Courts Differ in regard to Inhaling . . . . . . . . . . . . . . . 359
Girls who have Push . . . . . . . . 766
Glaucoma after Discission of Secondary Cataract . . . . . . . . . . 740
Gould, Dr. George M. Address of, at the Buffalo University Medical College Commencement . . . . . 682
Grip The, and The Law . . . . . . 488
Gross, Samuel D., M. D. Proposed Monument to . . . . . . . . . . 625
Gynecology and Abdominal Surgery, Section of at Pan-American Medical Congress . . . . . 627
Pronunciation of . . . . . . 686

HARTWIG, MARCELL, M. D. A Pleuro-Trocar . . . . . . 495
Hayd, Herman E., M. D., Faradic Coil in Gynecological Practice . . 465
Head Support, An Inexpensive . . . 667
Health Department of Buffalo . . . 487
Michigan State Board of . . . 687
Hemorrhage . . . . . . . . . . . . 717
Hepatitis and Gastro-Enteritis, Subacute . . . . . . . . . . . . . . . 522
Hernia, Femoral and Ventral in Women . . . . . . . . . . . . . . 421
Hill, John D., M. D. Memorial of . 536
Himmelsbach, George A., M. D. Acute General Peritonitis Treated by Large Doses of Morphia . . . . 142
Hip, Excision of . . . . . . . . . . 669
Hip Disease, Appliance for the Prevention of Deformity in . . 527
Treated by the Portable Traction Splint . . . . . . . . 607
Hoffman, Joseph, M. D. Relation of Imperfect Surgery to the Sequelæ of Pelvic and Abdominal Operations . . . . . . . . . . . 77
Hopkins, Henry R., M. D. The Prevention of Tuberculosis . . . . . 321

### Hospital Notes:

Hospital, Arnot-Ogden Memorial . . . . . . . . . 683
Buffalo General . . . . . 683
Children's . . . . . . . 743
Jennie Casseday Infirmary for Women . . . . 743
Toronto General . . . . 626
New York Post-Graduate . 441

Howard, Mrs., Gift of . . . . . . . 687

PAGE.
Hubbell, Alvin A., M. D. Affections of Sight from Injuries . . . 129
Progress in Ophthalmology . . . . . . . . . . 102, 356, 736
A New Ear Scissors . . . . . 163
Hudor Company . . . . . . . 306, 768
Hysterectomy, A Plea for Early . . . 483

ICHTHYOL for Dyspepsia and Cephalalgias . . . . . . . . . . 489
Idiosyncrasy in Administration of Anesthetics . . . . . . . . . . . 740

### Illustrations:

Contributions to Brain Surgery, John B. Roberts, M. D. Fig. 1. Fractured Cranium, Showing Point where Trephine was Applied to Outer Surface . . . 395
Fig. 2. Inner Surface of same Specimen . . . . . . . . . . 395
Fig. 3. Diagram Showing Relations of Brain Tumor . . . 403
Fig. 4. Diagram Showing Lateral View of Cerebral Convolution and Fissures, to aid in making description clear . . 405
Neuro-Topographical Bust . . . 494
New Club Foot Shoe . . . . . 361
New Ear Scissors . . . . . . . 163
Pleura-Trocar . . . . . . . . . . 495
Polyclinic Ophthalmoscope . . . 206
Portrait of Dr. Frank Hamilton Potter, facing p. . . . . . . . 53
Portrait of Dr. R. P. Bush, facing p. . . . . . . . . . . . 439
Portrait of William Dean Howells, facing p. . . . . . . . . 641
Pozzi, Treatise on Gynecology. Fig. 1 (151). Enucleation of Interstitial Myoma . . . . 631
Fig. 2 (255). Gastro-hysteropexy . . . . . . . . . . . . . 631
Stamps for Recording Cases. Chest and Abdomen . . . . . 253
Gynecological . . . . . . . . 254

Incontinence of Urine, Rhus-Aromatica in the Treatment of . . . . . 137
Influenza, Is it Spread by the Wind?. 52
As a Cause of Optic Neuritis . 102
Bacillus . . . . . . . . . . 677
Deaths from . . . . . . . . . 714
Edema of the Glottis following . . . . . . . . . . . . 107
Some After-Effects of the Recent . . . . . . . . . . . . 65
Ingraham, Henry D., M. D. Nerve Counterfeits of Uterine Disease . . 449

PAGE.

**Instruments, New:**

Club Foot Shoe . . . . . . . . 361
Ear Scissors . . . . . . . . . . 163
Polyclinic Ophthalmoscope . . . 206
Stamps for Recording Cases . . 253

Iron, Subcutaneous Injections of . . . 232
Is a Child Viable at Six and a half Months? . . . . . . . . . . . . 342

JACOBSON, NATHAN, M. D. Converging Lines of Medical and Surgical Treatment . . . . . . 8
Jackson, Edward, M. D. How to Use Mydriatics . . . . . . 149
An Ophthalmoscope for General Use . . . . . . . . . . 204
Jaros Hygienic Underwear . . . . . 348
Jefferson Medical College, New Buildings for . . . . . . . . . . 548
Johnson and Johnson . . . . . . . . 256

**Journalistic Notes:**

Albany Medical Annals . . . . 501
Alienist and Neurologist . . . . 239
American Gynecological Journal. 308
Anales Nacionel de Hygiene . . 115
Balneology and Dietary, Journal of . . . . . . . . . . . . . 239
Climatologist, The . . . . . . 115
Cosmopolitan Magazine . . . . . . . . . . . 64, 114, 128, 255, 511
Daily Medical News . . . . . . 240
Detroit Free Press . . . . . . 384
Doctors' Weekly . . . . . . . 444
Hot Springs Medical Journal . . 501
International Medical Magazine . 628
Ladies' Home Journal . 256, 442, 555
Medical Record, New York . . . 552
Medical Fortnightly . . . . 186, 441
National Medical Review . . . 681
New England Medical Monthly . 115
New York Journal of Gynecology and Obstetrics . . . . . 305
Ohio Medical Journal . . . . . 500
Sanitarian, The Texas . . . . . 500
Sanitary Era . . . . . . . . : . 384
Southern Medical Record . . . . 441
Practitioner . . . . . . . 441
Post-Graduate . . . . . . . . 501

KEEN, W. W., M. D., Four Operations for Appendicitis . 269
Kentucky and the Quacks . . . . . 624
Kidney, Removal of, in Disease . . . 335
Knee-Joint, Arthrectomy of . . . . . 1

PAGE.

Knee-Joints, Double Dislocation of . 513
Knee, Voluntary Subluxation of from Muscular Action . . . . . . . . 527
Knock-Knee, Atonic . . . . . . . . . 671
Krauss, William C., M. D. Rhus Aromatica in the Treatment of Incontinence of Urine . . 137
A Neuro-Topographical Bust . 493
A "Much Nervous" Case . . 586
Progress in Medicine . 51, 104, 489

LABOR, Intra-Uterine Irrigation after . . . . . . . . . . . . 337
Lachrymal Passages, Obstructive Disease of, and Associated Intra-Nasal Lesions . . . . . . . . . 593
Law Governing the Practice of Medicine in New York, and how it is Enforced . . . . . . . . . . . . 762
Life Insurance, Duties of Physicians as Examiners for . . . . . 69
and Death Certificates . . . . 616
Literary Theft . . . . . . . . . . 684
Litigants, Physical Examination of . 487
Lymph-Streams and Lymph-Channel of the Eye . . . . . . . . . 737

MACKENZIE, SIR MORELL, A Suit by . . . . . . . . . 102
A Monody on . . . . . . . 685
Malpractice and Morphine . . . . . 360
A Nebraska Case . . . . . . 360
A New York Case . . . . . 618
Case, A Famous . . . . . . 684
Manley, Thomas H., M. D. Report of a Case of Double Dislocation of the Knee-Joints . . . . . . . . . 513
Mays, Thomas J., M. D. Concentrated Food in the Treatment of Pulmonary Consumption . . 74
Sweet Oil in the Treatment of Gall-Stones . . . . . . . 207

**Medical College Notes:**

Buffalo University . . . . . . 115
Department of Pharmacy . 369
Chicago College of Physicians and Surgeons . . . 116, 240, 625
Convention of Medical Colleges in Ohio . . . . . . . . . . . 368
Harvard Medical School . . . . 444
Jefferson Medical College . 548, 627
Long Island College Hospital . . 117
Medico-Chirurgical College of Philadelphia . . . . . . . . . 117
Niagara University . . . . . . 116
Post-Graduate Medical School . 369
Tulane University . . . . . . 116

PAGE.
Medical Progress, Retrospect of in Erie County . . . . . . . . . . . 470
Medico-Legal and Medico-Ethical . . 619
Morton, Thomas, S. K., M. D. The Operative Treatment of Appendicitis . . . . . . . . . . . . . . 275
Metric Formulæ . . . . . . . . . 233
Miller, John A., Ph. D., Progress in Physiological Chemistry . . . 97, 349
Motion, Effect of Persistent . . . . 665
Mydriatics, How to Use . . . . . . 149
Mynter, Herman, M. D. Arthrectomy of the Knee-Joint . . 1
Senile Gangrene . . . . . . 457

NEEDLES, Prevention of Rust on Surgical . . . . . . . . 161
Nervous Case, A "Much" . . . . . 586
Neurosis, Three Cases of Electrical Traumatic . . . . . . . . . . . . 140
Neuro-Topographical Bust . . . . 493
New York Physicians' Mutual Aid Association . . . . . . . . . . 512, 745
Niagara University Medical College Commencement . . . . . . . . . 679
Notes and Comment . . . . . . . . 499

**Obituary:**

BROWN, JOSEPH B., M. D., Surgeon U. S. A. . . . . . 239
Campbell, Henry F., M. D. . . . 366
Chace, William, M. D. . . . . . 383
Clark, Simeon T., M. D. . . . . 383
Craig, James W., M. D. . . . . 113
Damainville, Lucien, M. D. . . 366
Hill, John D., M. D. . . . . . 556
Mackenzie, Sir Morell . . . . . 685
Potter, Dr. Frank Hamilton . 53, 164
Riley, Henry A., A. B., LL. B. . 750
Ross, James, M. D. . . . . . . 690
Warren, Mr. Orasmus G. . . . . 690

Obstetric Calendar . . . . . . . . 538
Obstetrical Record . . . . . . . . 319
Ohio Medical Practice Act . . . . . 624
Ointments and Pastes . . . . . . . 325
Operations, Effect of Per Se . . . . 161
Ophthalmia, Sympathetic, after Cataract Operations . . . . . . . . . . 737
Ophthalmoscope, An, for General Use . . . . . . . . . . . . . . 204
Opiates, Contra-Indications for the Use of . . . . . . . . . . . . . 581
Orthopedic Surgery, Indications for Operative Interference in . . . . 671
Ovarian Cysts, Suppurating . . . . . 480

PAGE.
PAN-AMERICAN Medical Congress Bulletins . . 444, 445, 569
Section on Medical Pedagogics 688
Papoid, or Vegetable Pepsin . . . . 628
Parmenter, John, M. D., Progress in Surgery . . . . . . . . . . . . 159
Parturition, Use of Chloroform in . . 649
Pastes and Ointments . . . . . . . 325
Patellæ, Congenital Dislocation of Both . . . . . . . . . . . . . 718
Pedagogics, Medical . . . . . . . . 688
Pelvic Abscess . . . . . . . . . . 481
Peritonitis, Case of Acute General Treated by Morphine . . . . . . 142
Perineum, Emmet's Operation for Laceration of . . . . . . . . . . 409

**Personals:**

Andrews, J. B., M. D. . . . . . 689
Ashton, W. E., M. D. . . . . . 747
Bailey, William C., M. D. . . . 184
Beaver, General James A. . . . 111
Bellamy, Russell, M. D. . . . . 110
Briggs, W. T., M. D. . . . . . 304
Bulkley, L. Duncan, M. D. . . 110
Bush, R. P., M. D. . . . . . . 439
Clark, Edward, M. D. . . . . . 238
Coles, Walter, M. D. . . . . . 501
Cullen, Gilbert I., M. D. . . . 689
Curran, Richard, M. D. . . . . 554
Darling, F. B., M. D. . . . . . 110
Davis, W. E. B., M. D. . . 304, 443
Dixon, Arch., M. D. . . . . . . 748
Eastman, Joseph, M. D. . . . . 57
Eddy, J. L., M. D. . . . . . . 629
Fisher, John C., M. D. . . . . . 555
Gibbons, Henry, Jr., M. D. . . . 238
Hamilton, John B., M. D. . . . 184
Harte, Miss Jessamy . . . . . 749
Heath, Richard A., M. D. . . . 501
Heath, William H., M. D. . . . 502
Howells, William Dean . . . . 748
Hubbell, Alvin A., M. D. 184, 629, 748
Hummel, A. L., M. D. . . . . 56
Johnston, Geo. Ben., M. D. . . . 367
Krauss, William C., M. D. . 501, 629
Lewis, Daniel, M. D. . . . . . 383
Mann, Matthew D., M. D. . . . 690
Martin, A. J., M. D. . . . . . . 747
Miller, John A., Ph. D. . . . . 110
Montgomery, E. E., M. D. . . . . . . . . . . . . . 110, 554, 629
Morris, Robert T., M. D. . . . 747
O'Hara, A. F., M. D. . . . . . 501
Ouchterlony, John A., M. D. 238, 555
Parmele, Mr. Charles Roome . . 56
Pettit, John A., M. D. . . . . . 305
Pilcher, Lewis S., M. D. . . . . 748
Pohlman, Julius, M. D. . . . . 184

PAGE.

Praeger, E. Arnold, M. D. . . . . 238
Price, Joseph, M. D. . . . . . . . 554
Putnam, W. A., M. D. . . . . . . 367
Rauch, John H., M. D. . . . . . 111
Reed, Charles A. L., M. D. . . . 305
Rohé, George H., M. D. . . . . . 110
Ross, James F. W., M. D. . . . . 555
Ross, T. Haven, M. D. . . . . . 748
Sanders, Nelson, M. D. . . . . . 367
Southard, William B., M. D. . . 502
Stanton, Byron, M. D. . . . . . . 502
Stockton, Charles G., M. D. . . 749
Thornbury, F. J., M. D. . . . . . 629
Ward, M. B., M. D. . . . . . . . 238
Walker, Dr. Edwin . . . . . . . 56
Wende, Ernest, M. D. . . . . . . 304
Whelpley, H. M., M. D. . . . . . 110
Wheeler, William A., M. D. . . 305

Petrolatum . . . . . . . . . . . . . 153
Pharmaceutical Congress . . . . . . 686
Phthisis, Potassium Iodide in the Diagnosis of . . . . . . . . . . 648
Physicians. Must They Answer Calls? . . . . . . . . . . . . . . 100
Physiological Chemistry . . . . . . 349
Pilocarpine in Labyrinthine Disease . . . . . . . . . . . . . . . 144
Plaster Casts of Thorax in Rotary Lateral Curvature . . . . . . . . 532
Pneumonia and its Treatment . . . . 656
Post-Partum Hemorrhage . . . . . . 333
Post-Master General, Annual Report of . . . . . . . . . . . . . . . 499
Potter, Frank Hamilton, M. D. Action of Medical Society County of Erie in Relation to Death of . . . . . . 37
Action of Faculty of Niagara University . . . . . . . . 44
of Buffalo University . . . 48
American Laryngological Society . . . . . . . . 176
Comments of Medical and Newspaper Press on death of . . . . . . . . . . . 164
Memorial of the Thursday Club . . . . . . . . . . . 49
Civil Service Reform Association . . . . . . . . 177
Some After-Effects of the Recent Influenza, by . . . . . 65
Pregnancy, Lactation in . . . . . . 580
Presentations, Transverse, A New Method of Treatment in . . . . . 385
Price, Joseph, M. D., Report of Cases of Appendicitis . . . . . . 258
Gynecological Work of . . . . 676
Prize, The Alvarenga . . . . . . . 440
Prizes, Pope Manufacturing Co. . . 576

PAGE.

**Progress in Medical Science:**

Medicine . . . . . . . . 51, 104, 489
Medical Jurisprudence . . . . . . . . . . . . . 99, 358, 487, 616
Ophthalmology . . . . 102, 356, 736
Physiological Chemistry . . . 97, 349
Surgery . . . . . . . . . . . . 159
Therapeutics . . . . . . . . 233, 539

Proteids, Pathology of . . . . . . . 98
Pustule, Nitric Acid in Malignant . . 675
Putnam, James W., M. D. Three Cases of Electrical Traumatic Neurosis . . . . . . . . . . . . . . . 140

RECTUS TENDON, Operation for Advancement of . . . . . 357

**Reviews:**

Abbott: Principles of Bacteriology . . . . . . . . . . . . 568
Adams, F.: Genuine Works of Hippocrates . . . . . . . . . 315
Adams, W.: Electricity . . . 316
Ball: Essentials of Bacteriology 376
Bartholow: Manual of Hypodermatic Medication . . . . 633
Beard: Medical and Surgical Uses of Electricity . . . . . 307
Billings: Index-Catalogue, Surgeon-General's Office . . . . 242
Bigelow: Electricity and Batteries . . . . . . . . . . . . 122
Brockway: Essentials of Physics . . . . . . . . . . . . . 699
Brubaker: Human Physiology . 379
Canfield: Urinary Analysis . . 376
Carpenter: Revelations of the Microscope . . . . . . . . . 507
Chauveau: Anatomy of Domesticated Animals . . . . . . . 374
Childs: Public Ledger Almanac 569
Coltman: The Chinese . . . 637
Culver and Hayden: Manual of Venereal Diseases . . . . . 632
Curtman: Uses, Tests, etc., of Chemical Re-Agents . . . . 251
Davis: Manual of Obstetrics . 377
Davis: Hand-Book on Potable Water . . . . . . . . . . . 250
Davis: Consumption . . . . . 698
Foster: Text-Book of Physiology . . . . . . . . . . . . 566
Fraenkel: Text-Book of Bacteriology . . . . . . . . . . 243
Gage: Microscope and Histology . . . . . . . . . . . . 375

PAGE.
Gibbes: Pathology and Morbid Histology . . . . . . . . . 370
Grandin: Electricity in Gynecology . . . . . . . . . . . 311
Hamilton: Lectures on Tumors . . . . . . . . . . . 248, 760
Hammond: Diseases of the Nervous System . . . . . . 62
Hare: Essentials of Physiology 378
Hare: System of Therapeutics 564
Hare: Fever and Antipyretics . 119
Hare and Martin: Wounds of Intestines . . . . . . . . . 378
Helbing: Modern Materia Medica . . . . . . . . . . . . . 379
Huidekoper: Age of Domestic Animals . . . . . . . . . . 505
Hurd: Insomnia and Hypnotics 638
Jaworski: Carlsbad Sprudel Salt . . . . . . . . . . . . . 122
Keating: International Clinics . . . . . . . . . . . . 61, 567
King: Stories of a Country Doctor . . . . . . . . . . 313
Leonard: Pocket Anatomist . . 121
Lewis: Cancer and its Treatment . . . . . . . . . . . 700
Long: Tables for Doctor and Druggist . . . . . . . . . 250
Love: Diseases of Children . . 118
Macfarlane: Insomnia and its Therapeutics . . . . . . . . 309
McClellan: Regional Anatomy 446
Mays: Pulmonary Consumption 502
Moullin: A Practical Treatise on Surgery . . . . . . . . 310
Muter: Manual of Chemistry . 372
Nancrede: Essentials of Anatomy . . . . . . . . . . . 314
Nissen: Swedish Educational Gymnastics . . . . . . . . 506
Norris: Syllabus of Obstetrical Lectures . . . . . . . . . 313
Osler: Principles and Practice of Medicine . . . . . . . . . 754
Ostrom: Massage and Swedish movements . . . . . . . . . 504
Park: Mütter Lectures on Surgical Pathology . . . . . . . 697
Powell: Pocket Medical Formulary . . . . . . . . . . . . 698
Post: Massage . . . . . . . 316
Pozzi: Treatise on Gynecology . . . . . . . . . . . . 630, 695
Pye: Surgical Handicraft . . . 759
Reese: Text-Book of Medical Jurisprudence and Toxicology 245
Remondino: History of Circumcision . . . . . . . . . . 757
Rohé: Text-Book of Hygiene 60

PAGE.
Rohé: Manual of Diseases of the Skin . . . . . . . . 635
Roosa: Treatise on Diseases of the Ear . . . . . . . . . . 562
Sajous: Annual of the Universal Medical Sciences . . . 188, 750
Schultze: Lehrbuch der Hebammenkunst . . . . . . . . . 699
Seifert: Manual of Clinical Diagnosis . . . . . . . . . 189
Senn: Principles of Surgery . . 187
Surgical Bacteriology . . . 244
Sexton: Deafness . . . . . . 700
Shakespeare: Report on Cholera in Europe and India . . . 751
Shaw: Essentials of Nervous Disease and Insanity . . . . 508
Shoemaker: Materia Medica and Therapeutics . . . . 59
Heredity, Health, and Personal Beauty . . . . . . 248
Stewart: Essentials of Medical Electricity . . . . . . . . . 508
Stillman: Treatment of Chronic Articular Osteitis of the Hip . 636
Sozinskey: Medical Symbolism . 568
Taylor: Practice of Medicine . 117
Thomas: Treatise on Diseases of Women . . . . . . . . . 557
Thornton: Origin, etc., of Man . . . . . . . . . . . . 120
Three Thousand Questions on Medical Subjects . . . . . . 379
Transactions American Gynecological Society . . . . . 120
American Association of Obstetricians and Gynecologists . . . . . . . . 760
American Surgical Association . . . . . . . . 758
Association of American Physicians . . . . . 121, 634
Medical Society of the State of New York . . . . . 241
National Association of Railway Surgeons . . . . 761
Southern Surgical and Gynecological Association . . 246
Treves: Manual of Operative Surgery . . . . . . . . . . 692
Tyson: Practical Examination of the Urine . . . . . . . . 123
Vierordt: Text-Book of Diagnosis . . . . . . . . . . . 373
Wharton: Minor Surgery and Bandaging . . . . . . . . . . 247
Whelpley: Chemical Lecture Notes . . . . . . . . . . . 315
Whitta: Dictionary of Treatment . . . . . . . . . . . 700

PAGE.

Willard: Anesthesia and Anesthetics . . . . . . . . . . . 377
Williams: International Medical Annual . . . . . . . . 696
Wollen: Mothers' Hand-Book 249
Wood: Eye Diseases . . . . 699
Wood: Therapeutics . . . . . 371
Wood's Medical and Surgical Monographs . . . . . . 188, 560
Wyman: Diseases of the Bladder and Prostate . . . . . . 636
Visiting List, Medical News . . 312
Visiting List, Blakiston's . . . . 315
Year-Book of Treatment for 1892, Lea Brothers & Co. . . 637

Rheumatism, Application for . . . . 540
Ribs, Necrosis of, Complicating Pott's Disease . . . . . . . . . . . 19
Riley, Henry A., A. B., LL. B., Medical Jurisprudence . 99, 358, 487, 616

SACRAL RESECTION, Application of, to Gynecological Work . . . . . . . . . . . . 343
Scoliosis, Does it ever give rise to Pressure Myelitis? . . . . . . . 529
Self-Medication, The Prevalent Fashion of . . . . . . . . . . . . . . 521
Shock, Treatment of . . . . . . . 540
Sight, Affections of from Injuries . . 129
Skull, Pistol-Shot Wounds of . . . . 162
Smith, S. MacCuen, M. D. Employment of Pilocarpine in Labyrinthine Disease . . . . . . . . . 144

**Society Meetings:**

American Society of Microscopists . . . . . . . . . 57
Academy of Medicine . . . 691
Association of Obstetricians and Gynecologists . . 112, 691
Dermatological Association . 58
Electro-Therapeutic Association . . . . . . 113, 691
Medical Association . . . . 556
National Association of Military Surgeons, U. S. N. G. 370
Neurological Association . . . . . . . . . . . . . . 57, 749
Pharmaceutical Association . 691
Public Health Association . 111
Buffalo Clinical Society . . . . 306
Canadian Medical Association . 113
Congress of American Physicians and Surgeons . . . . . . . . 185
District Medical Societies of Northern Ohio . . . . . . 185

PAGE.

International Congress of Obstetrics and Gynecology . 557
Medical Congress, Eleventh, 691
Medical Association of Central New York . . . . . . . . . 749
Medical Society of the County of Erie . . . . . . . . . . . 692
Medical Society of the State of New York . . . . . 306, 443, 691
Mississippi Valley Medical Association . . . . . . . . . 112, 691
Orthopedic Association . . 113, 220
Pan-American Medical Congress, 556
Railway Surgeons, New York State Society of . . . . . . . 185
Southern Surgical and Gynecological Association . . . . . 241
Tri-State Medical Association . . 112
Western Society of Obstetricians and Gynecologists . . . . . 500

**Society Proceedings:**

American Association of Obstetricians and Gynecologists 333, 407, 475
Buffalo Medical and Surgical Association . . . 35, 94, 156, 228
Harlem Medical Association . . 431
Jefferson County (Alabama) Medical Society . . . . . . . . 89
Medical Society of the County of Chautauqua . . . . . . . . 96
Medical Society of the County of Erie . . . . . . . 37, 424, 532
Medical Society of the County of Niagara . . . . . . . . . . 484
New York Academy of Medicine —Section on Orthopedic Surgery . . . . 22, 333, 407, 475, 524, . . . . . . . . . . . 606, 665, 718
Philadelphia County Medical Society . 30, 77, 144, 204, 593, 656

Somnal—A Hypnotic . . . . . . . 105
Southern Pines . . . . . . . . . . 575
Spina Bifida Obstructing Labor . . . 434
Stamps for Recording Cases . . . . 253
State Medical Examining and Licensing Board . . . . . . . 179, 688, 762
Stone, R. French, M. D. Biography of American Physicians . . . . . 553
Stormont Medical Library . . . . . 684
Strontium, Bromide of . . . . . . . 717
In Gastric Troubles . . . . . 678
Student's Exemption Bill . . . . 552, 687
Sulfonal, Modifications of Pulse in use of . . . . . . . . . . . . . . . 490
Surgery, Relation of Imperfect, to the Sequelæ of Pelvic and Abdominal Operations . . . . . . . . . 77
Syphilodermata, Peculiar Case of . . 330

PAGE.

TAIT, Denholm versus . . . . . 619
Therapeutic Notes . . . . . . 539
Thornbury, Frank J., M. D. Letter from . . . . . . . . . . 543
Inert Tubercle Bacilli . . . . 654
Bacteria of the Mouth . . . . 715
Tic Douloureux, Pathological Anatomy of . . . . . . . . . . . 105
Topics of the Month . . . . . . . 681
Treatment, Converging Lines of Medical and Surgical . . . . . . 8
Trendelenburg's Posture in Gynecology . . . . . . . . . . . . 479
Trephining, Indications for . . . . 159
Trespassing, Novel Punishment for . 360
Tuberculosis, New Remedies Against, 104
The Prevention of . . . . . . 321
Tubercle Bacilli, Inert . . . . . . . 654
Tuberculin . . . . . . . . . . . . . 349
Truax, Greene & Co., and the World's Fair . . . . . . . . . . 627

UTERUS, Shall we use the Uterine Sound to Correct Backward Displacements of? . . 201

PAGE.

Uterus, Operative Treatment of Retro Displacements of . . . 577
Uterine Appendages, Removal of, with Results . . . . . . . 407
Disease, Nerve Counterfeits of, 449
Unique Case, A . . . . . . . . . . 492

VOLUME XXXI., Close of . . 746

WEIGEL, LOUIS A., M. D. Necrosis of the Ribs, Complicating Potts' Disease . . 19
Wende, Ernest, M. D. Ointments and Pastes . . . . . . . . . . . 325
Werner, Marie B., M. D. Some Contra-Indications for the Use of Opiates . . . . . . . . . . . . . 581
Wounds of Abdomen . . . . . . . 160
of Skull . . . . . . . . . . 162
Wright, Adam H., M. D. The General and Special Medical Societies of America . . . . . . . . . . 194

